# Port delivery system with ranibizumab (Susvimo) recall- What does it mean to the retina specialists

**DOI:** 10.1186/s40942-023-00446-z

**Published:** 2023-01-30

**Authors:** Ashish Sharma, Arshad M. Khanani, Nikulaa Parachuri, Nilesh Kumar, Francesco Bandello, Baruch D. Kuppermann

**Affiliations:** 1 Lotus Eye Hospital and Institute, Avinashi Road, Coimbatore, 641014 TN India; 2 Sierra Eye Associates, The University of Nevada, Reno School of Medicine, NV Reno, USA; 3 Department of Vitreoretina, Sankara Eye Hospital, Coimbatore, TN India; 4 Madhavi Netralaya, Ara, Bihar India; 5 University Vita-Salute, Scientific Institute San Raffaele, Milan, Italy; 6 Gavin Herbert Eye Institute, University of California, Irvine, CA USA

**Keywords:** Susvimo, Port Delivery System, Ranibizumab, Septum: anti-VEGF, Recall

Port delivery system (PDS) with ranibizumab (Susvimo, Genentech, USA) was approved by the U.S. Food and Drug Administration (FDA) on 22nd October 2021 for the management of neovascular age-related macular degeneration (n-AMD) in eyes with at least two prior anti-vascular endothelial growth factor (VEGF) injections [1, 2]. Sustained release drug delivery for the management of retinal vascular diseases has been a long-term desire of retinal physicians across the globe and Susvimo has provided the same with its innovative implants design and high concentration ranibizumab formulation (100 mg/ml) after years of research and development [3]. Approval of Susvimo was based on the phase 3 Archway study for eyes with previously treated n-AMD [1]. Susvimo is also being evaluated for the management of diabetic macular edema (DME) in PAGODA phase 3 trial and for diabetic retinopathy in PAVILION phase 3 trial [1]. Susvimo had gone through various challenges during its development processes. High rates of vitreous hemorrhage were seen in the early part of the phase 2 Ladder study where 179 patients received the PDS implant [4]. This led to the initial pause and modification in the surgical procedure to mitigate the issue. This drastically improved the incidence of vitreous hemorrhage in the later part of phase 2 and future trials. In the phase 3 Archway study, 248 patients with nAMD received treatment with the PDS implants, there were 4 (1.6%) endophthalmitis cases, 2 (0.8%) retinal detachments, 13 (5.2%) vitreous hemorrhages, 6 (2.4%) conjunctival erosions, and 5 (2.0%) conjunctival retractions [2]. The procedure has continued to evolve even after Archway as it was noticed that most of the adverse events were technique related and majority due improper closure of conjunctiva and tenon’s capsule and this has led to meticulous training of retinal surgeons regarding the technique [5]. Hopefully, improved surgical techniques and proper surgery training will lead to lower rates of adverse events in the ongoing PAGODA and PAVILION studies. In October 2022, Genentech announced the recall of implant. This brief manuscript will discuss the details of the recall and what it means to retinal physicians in the long term.

## What is being recalled and what is not

The recall included Susvimo ocular implant and insertion tool assembly, including the Susvimo (ranibizumab) drug vial and initial fill needle (lot numbers 3499188 and 3523071) which are sold together. However, it did not include the Susvimo (ranibizumab injection) 100 mg/ml drug vial for a refill-exchange solution and refill needles in order to allow for continued refill-exchange procedures in eligible patients in clinical trials as well as in the commercial setting who already have an implant. Furthermore, this recall will not include the Susvimo explant tool. New commercial implantations as well as implantations for patients in ongoing clinical trials have been stopped [6].

## Reason for recall

In the Archway and Portal studies, patients with PDS received fixed refills every 6 months. This was in contrast to prn (as needed) refills in the phase 2 Ladder study, where the median time to refill was 15.8 months. The control arm in phase 2 and 3 studies was monthly ranibizumab and data from both studies show that PDS significantly reduces the treatment burden compared to monthly injections. Therefore, PDS can be clinically beneficial by decreasing treatment burden for patients with nAMD, especially the high need patients requiring frequent injections. Cases of septum dislodgement in implants was recognized in patients with the device after refill-exchange procedures in the Archway phase 3 trial and the Portal extension study. The cases of septum dislodgement in the phase 3 trial initiated additional quality testing of the septum with repeated puncturing of the implant with refill needle to further investigate the performance of the septum over the long term. During such testing, it was found that many implants did not perform as per the quality standard and this led to the pause on all implantations, both in the study and commercial settings until the quality issue is rectified [6].

## Rate of septum dislodgement

As of August 31, 2022, 33 reported cases of septum dislodgement have been reported in 1419 patients with implants (2.3%, including re-implantations) and during 5236 refill-exchange procedures (0.63%) across all PDS clinical trials. No patients with the implant from the phase 2 Ladder trial have resulted in septum dislodgement. Furthermore, no patient in the commercial setting has had septum dislodgement as of September 30, 2022 [6].

## How to identify septum dislodgement and its impact

For many patients who are already on Susvimo, it is important for the physicians to do a careful dilated retinal examination and check all the parts of the implants to determine if they are in the proper place. To examine the septum specifically, they should see whether there is any tilt at the septum which is normally perpendicular in an intact implant. Septum dislodgement could potentially disturb the functional mechanism of the implant and refill-exchange procedures will not be able to be performed.

## Recommendations for physicians

Patients who already have the Susvimo implant can continue to get refill-exchanges if there is no septum dislodgement. However, in patients in which septum dislodgement is identified, a decision to leave or remove the implant needs to be made after a mutual discussion between the treating physician and the patient. In both these cases, patients can be treated with regular intravitreal anti-VEGF injections at the discretion of the physician. At this time, exchanging the implant is not an option as all the new implant procedures are on hold.

## What does it mean to industry and physicians going forward?

Voluntarily recalling a product is to be taken as a responsible action from the industry because patient safety is of utmost priority for regulatory authorities. Any innovation has challenges and Susvimo has successfully overcome some challenges in the past such as a high rate of vitreous hemorrhage compared to intravitreal therapy. The question now is whether the septum problem can be rectified and, if so, will physicians resume using it. The answer to this is not known yet but it will require physicians and patients alike to weigh risks of undergoing surgery and having an implant in the eye compared to well-known long term benefits of sustained delivery with Susvimo. Unlike the brolucizmuab inflammation safety issues which cannot mitigated [7], it is hoped that since Susvimo has undergone a voluntary recall and it is related to quality testing, this will be rectified in the near future and implant will be again available for patients.

To summarize, the port delivery system with ranibizumab is a transformational next step in the field of retinal pharmacotherapy and is a proven, highly-effective long-term sustained release drug delivery system for anti-VEGF therapy [7]. Hopefully, the quality issues related to septum dislodgement issue will be resolved in the near future and allow for this innovative form of therapy to be available once again for patients with nAMD and possibly other retinal diseases.

## Data Availability

Not applicable**.**

## References

[1] FDA Approves Genentech’s Susvimo, a first-of-its-kind therapeutic approach for wet age-related macular degeneration (AMD). 2022 https://www.gene.com/media/press-releases/14935/2021-10-22/fda-approves-genentechs-susvimo-a-first-. Accessed 8 Dec 2022.

[2] Holekamp NM, Campochiaro PA, Chang MA, Miller D, Pieramici D, Adamis AP, Brittain C, Evans E, Kaufman D, Maass KF, Patel S, Ranade S, Singh N, Barteselli G, Regillo C, all Archway Investigators (2022). Archway randomized phase 3 trial of the port delivery system with ranibizumab for neovascular age-related macular degeneration. Ophthalmology.

[3] Sharma A, Kumar N, Parachuri N, Kuppermann BD, Bandello F, Regillo CD (2020). Ranibizumab port delivery system (RPDS): realising long awaited dream of prolonged VEGF suppression. Eye (Lond).

[4] Khanani AM, Callanan D, Dreyer R, Chen S, Howard JG, Hopkins JJ, Lin CY, Lorenz-Candlin M, Makadia S, Patel S, Tam T, Gune S, of the Ladder Investigators (2021). End-of-study results for the ladder phase 2 trial of the port delivery system with ranibizumab for neovascular age-related macular degeneration. Ophthalmol Retina.

[5] Sharma A, Parachuri N, Kumar N, Kuppermann BD, Bandello F (2022). The port delivery system with ranibizumab-journey of mitigating vitreous hemorrhage. Eye (Lond).

[6] Voluntary recall of the SUSVIMOTM Ocular Implant. 2022. https://www.gene.com/download/pdf/Susvimo_DHCP_Important_Prescribing_Information_2022-10-18.pdf. Accessed 8 Dec 2022.

[7] Sharma A, Kumar N, Parachuri N, Sharma R, Bandello F, Kuppermann BD, Loewenstein A (2020). Brolucizumab and immunogenicity. Eye (Lond).

